# Screening and assessment of malnutrition in patients with liver cirrhosis

**DOI:** 10.3389/fnut.2024.1398690

**Published:** 2024-07-18

**Authors:** Yumei He, Zhiming Wang, Shiyan Wu, Lu Li, Jiazhen Li, Yexing Zhang, Boshi Chen, Xiaobin Sun, Chao Sun, Liping Wu

**Affiliations:** ^1^North Sichuan Medical College, Nanchong, China; ^2^Department of Gastroenterology, The Third People’s Hospital of Chengdu, The Affiliated Hospital of Southwest Jiaotong University, Chengdu, China; ^3^Department of Gastroenterology, The Affiliated Hospital of Southwest Medical University, Luzhou, China; ^4^Department of Clinical Nutrition, The Third People’s Hospital of Chengdu, The Affiliated Hospital of Southwest Jiaotong University, Chengdu, China; ^5^Department of Gastroenterology and Hepatology, Tianjin Medical University General Hospital, Tianjin, China

**Keywords:** cirrhosis, risk assessment, assessment, malnutrition, prevalence

## Abstract

The development and advancement of malnutrition is associated not only with the progression of hepatic dysfunction, but also with cirrhosis-related complications. However, the prevalence of malnutrition reported in different studies varies widely due to differences in diagnostic methods and patient investigation settings. Therefore, we need to identify malnourished patients promptly and accurately. The purpose of this review was to compare the validity and reliability of nutritional screening tools and to select the most appropriate nutritional risk screening for patients with cirrhosis. We compared nutritional risk screening tools such as the Nutritional Risk Screening 2002 (NRS-2002), Malnutrition Universal Screening Tool (MUST), Royal Free Hospital-Nutritional Prioritizing Tool (RFH-NPT) and Liver Disease Undernutrition Screening Tool (LDUST). Royal Free Hospital-Nutritional Prioritizing Tool (RFH-NPT) is more feasible to screen cirrhotic patients for nutritional risk, and is highly reproducible, considering the impact of sodium and water retention; so it is practical to screen cirrhotic patients via RFH-NPT for nutritional risk, subsequently, to evaluate the nutritional status of patients with nutritional risk via the Global Leadership Initiative on Malnutrition (GLIM) diagnostic criteria. L3-SMI (third lumbar-skeletal muscle index) can accurately define sarcopenia in cirrhotic patients and also be used for clinical nutritional status assessment.

## 1 Introduction

Malnutrition is a frequent complication of liver cirrhosis and closely correlated with poor prognosis, especially in patients with decompensated cirrhosis (1); It is defined as changes in mental and physical functioning due to alterations in body composition and cellular quality, leading to poor clinical outcomes and reduced quality of life (2). The prevalence of malnutrition was 46 and 95% in Child-Turcotte-Pugh (CTP) A and C, respectively (3). The existing literature suggests that the prevalence of malnutrition in patients with decompensated cirrhosis may exceed 50% (4, 5).

**Figure 1:**
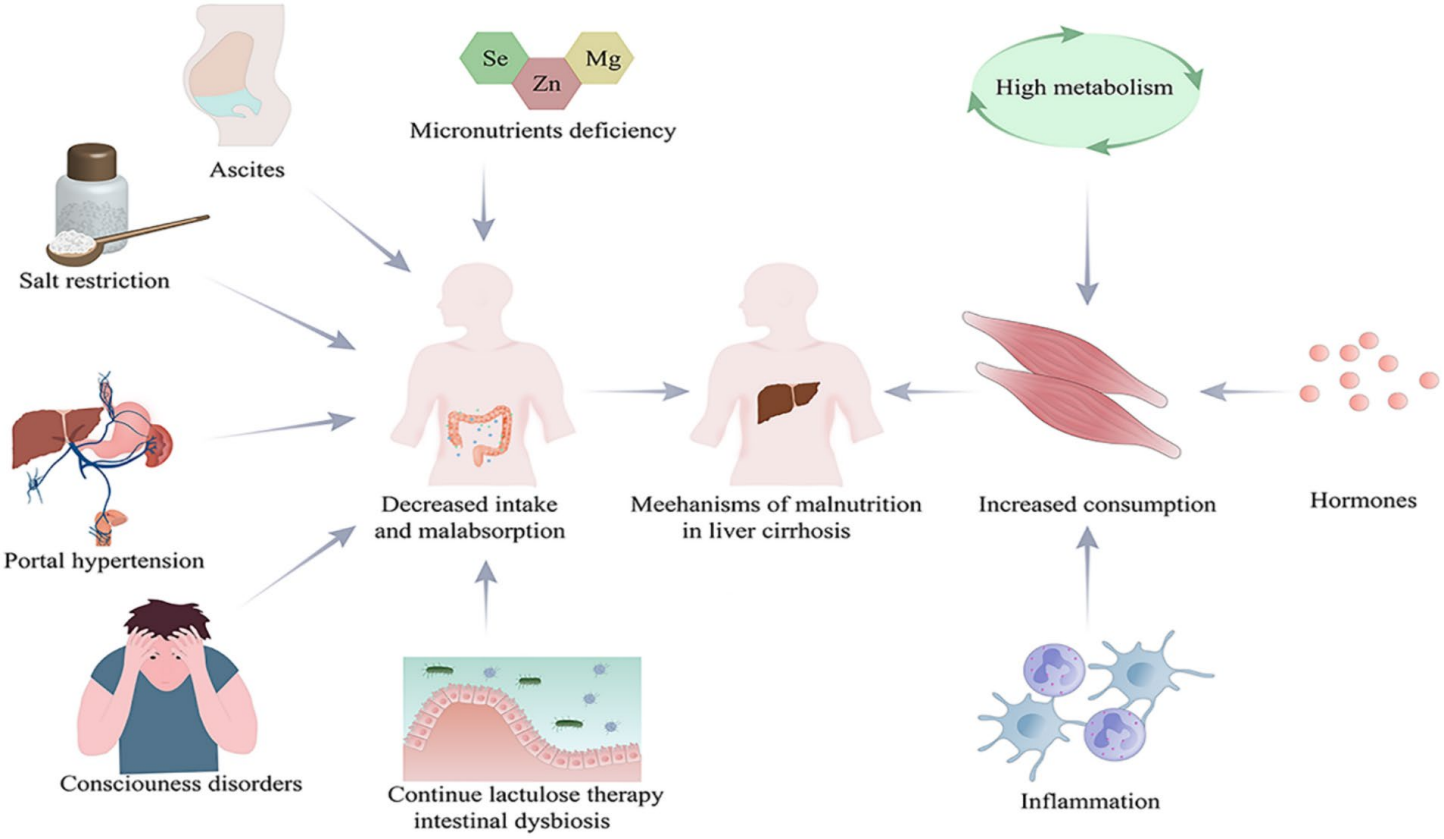
Causes of malnutrition in patients with cirrhosis. Created with Adobe Illustrator; Se, selenium; Zn, zinc; Mg, magnesium.

The causes of malnutrition in cirrhotic patients can be categorized into two main aspects, namely decreased intake and increased consumption (Figure 1). Decreased intake includes: (1) Loss of appetite, early satiety and impaired consciousness leading to reduced intake serve as the most common causes (6, 7). Patients with cirrhosis are usually deficient in micronutrients. Several studies have shown that serum levels of zinc, selenium, and magnesium are significantly low in patients with cirrhosis and decrease dramatically in correspondence with the disease progression (8). This may partially account for the loss of appetite in these patients (9). In addition, diets which restrict sodium may result in unpalatable food and may be a contributing factor to inadequate nutrient intake. Moreover patients with cirrhosis usually have ascites and portal hypertension, leading to slowed bowel movements and limited gastric diastole, which may result in delayed feeling of hunger and reduction of food intake (10). On the other hand, some cirrhotic patients with impaired consciousness due to hepatic encephalopathy are primarily dependent on parenteral nutrition, giving rise to inadequate nutrient supply. (2) Continuous lactulose therapy and dysbiosis of intestinal flora may lead to malabsorption (11). Studies have shown that the development of malnutrition in patients with cirrhosis is associated with dysbiosis of the intestinal microbiota. There is an increase in pro-inflammatory flora such as Enterobacteriaceae, a phenomenon that usually leads to inflammation in cirrhotic patients, accompanied with increased protein metabolism and loss of muscle mass (12). Resting energy expenditure accounts for 60–70% of total energy expenditure in healthy individuals (13) and is often increased in patients with cirrhosis due to hypermetabolism, inflammatory response, and immunosuppression (14). Patients with cirrhosis tend to have increased protein metabolism and decreased synthesis in close relation to malnutrition (15). Hormonal mediation of malnutrition is complex, and it includes the major orexigenic (appetite) hormone, gastrin, as well as a variety of anorexigenic (satiety) hormones, including leptin, cholecystokinin, glucagon-like peptide-1, peptide YY, oxyntomodulin, and pancreatic polypeptide (16, 17). It seems unclear that how inflammation or hormones affect nutrition consumption. In addition, hyperammonemia appears to be one of the important causes of protein depletion in cirrhosis as well (18).

The development and advancement of malnutrition is associated not only with the progression of hepatic dysfunction, but also with cirrhosis-related complications, including infections, hepatic encephalopathy, and ascites (19, 20). Decompensated cirrhosis often presents with severe ascites and portal hypertension, which is particularly detrimental to oral nutrition. A negative balance of calories and protein can further deteriorate the already impaired synthetic function in cirrhotic patients (21). Furthermore, malnutrition independently serves as a prognostic indicator for mortality (20). It is imperative to properly identify malnourished subjects with the purpose of providing appropriate treatment to improve the prognosis. The estimated prevalence of malnutrition in patients with cirrhosis ranges from 5–92% due to considerable variation in the measuring tools (7). Table 1 summarizes studies concerning the prevalence of malnutrition in the most recent publications by using different nutritional assessment tool. To standardize and harmonize the diagnosis of malnutrition, the Global Leadership Initiative on Malnutrition (GLIM) reached a new global consensus on the criteria for the diagnosis of malnutrition in 2019, a two-step modality for nutritional assessment is recommended, that is, risk screening of subjects using validated tools prior to diagnostic assessment and intervention, and provide diagnostic criteria for malnutrition according to phenotypic and etiologic parameters (33). The definition of nutritional risk screening varies slightly from a variety of organizations, with the American Dietetic Association’s Nutrition Care Process considering nutritional risk screening to be “those preventive services that use tests or standardized screening procedures to identify patients in need of specific interventions” (34). The American Society for Parenteral and Enteral Nutrition defines nutritional risk screening as “the process of identifying individuals who are malnourished or at risk of malnutrition in order to determine the need for a detailed nutritional assessment” (35). The European Society for Parenteral and Enteral Nutrition (ESPEN) states that nutritional risk screening is “a quick and simple process carried out by a medical practitioner,” while ESPEN provides a more global definition (36). When selecting a nutritional risk screening tool, we judged the ability of the tool by sensitivity, specificity, negative predictive value, and positive predictive value; we also needed to consider the feasibility of the screening tool, as overly time-consuming or complex screening tools are likely to result in a lower rate of accurate completion (37).

**Table 1:** Summary of studies showing the reported prevalence of malnutrition in patients with cirrhosis.

Authors, Year	Study population	Measure-ment tool	Prevalence of malnutrition (%)	Results summary
Oliveira et al., 2020 (22)	90 patients with cirrhosis	SGA PA	59.1 53.3	PA was a good method to assess prognosis.
Santos et al., 2022 (23)	152 patients with cirrhosis awaiting a liver transplant	SGA GLIM	63.2 0.7–30.9	The majority of GLIM combinations had poor agreement with SGA.
Chaney et al., 2020 (24)	134 patients with cirrhosis	SGA	47.8	Early treatment of malnourished patients with cirrhosis may reduce morbidity and LOS prior to transplantation.
Zambrano et al., 2020 (25)	118 patients with cirrhosis	PG-SGA	35.0	PG-SGA can be considered a good marker of sarcopenia that can be used in clinical practice.
Casas-Deza et al., 2023 (26)	57 clinically significant portal hypertension patients	LDUST	54.4	The LDUST has a solid ability to predict complications in cirrhosis outpatients with CSPH.
Topan et al., 2022 (1)	156 patients with cirrhosis	SGA RFH-NPT SMI MUAC	64.7 49.3 69.2 48.0	The combination between RFH-NPT and MUAC can be used as a valuable tool in daily practice.
Koulentaki et al., 2022 (27)	137 patients with cirrhosis	SGA MNA	60.0 43%	MNA was a strong predictor of mortality.
Javaid et al., 2022 (28)	83 patients with cirrhosis	SGA	88	Providing individualized nutritional intervention prevents further risk of malnutrition and related complications.
Wu et al., 2020 (29)	104 patients with cirrhosis	NRS-2002 RFH-NPT MUST LDUST SGA	51.0 63.2 38.1 70.3 63.0	The RFH-NPT was better able to predict the risk of malnutrition in patients with cirrhosis and had a superior prognostic value.
Boulhosa et al., 2020 (30)	166 patients with cirrhosis	NRS-2002 RFH-NPT GLIM	36.1 52.4 57.3	RFH-NPT has substantial agreement in identifying nutritional risk, good sensitivity and good value for diagnosing malnutrition in patients with advanced chronic liver disease.
Wang et al., 2022 (31)	135 patients with cirrhosis	RFH-NPT	65.2	Immune dysfunction measured by NLR was associated with malnutrition risk estimated by RFH-NPT in cirrhosis.
Yang et al., 2023 (32)	363 patients with decompensated cirrhosis	RFH-NPT GLIM	70.8 33.3	GLIM criteria may serve a specific proxy to diagnose malnutrition along with RFH-NPT screening.

SGA, Subjective Global Assessment; PA, phase angle; GLIM, Global Leadership Initiative on Malnutrition; PG-SGA, Patient-Generated Subjective Global Assessment; LOS, length of hospital stay; LDUST, Liver Disease Undernutrition Screening Tool; NRS-2002, Nutritional Risk Screening 2002; RFH-NPT, Royal Free Hospital-Nutritional Prioritizing Tool; SMI, Skeletal Muscle Index; MUAC, mid-upper arm circumference; CSPH, clinically significant portal hypertension; MNA, mini nutritional assessment.

**Figure 2:**
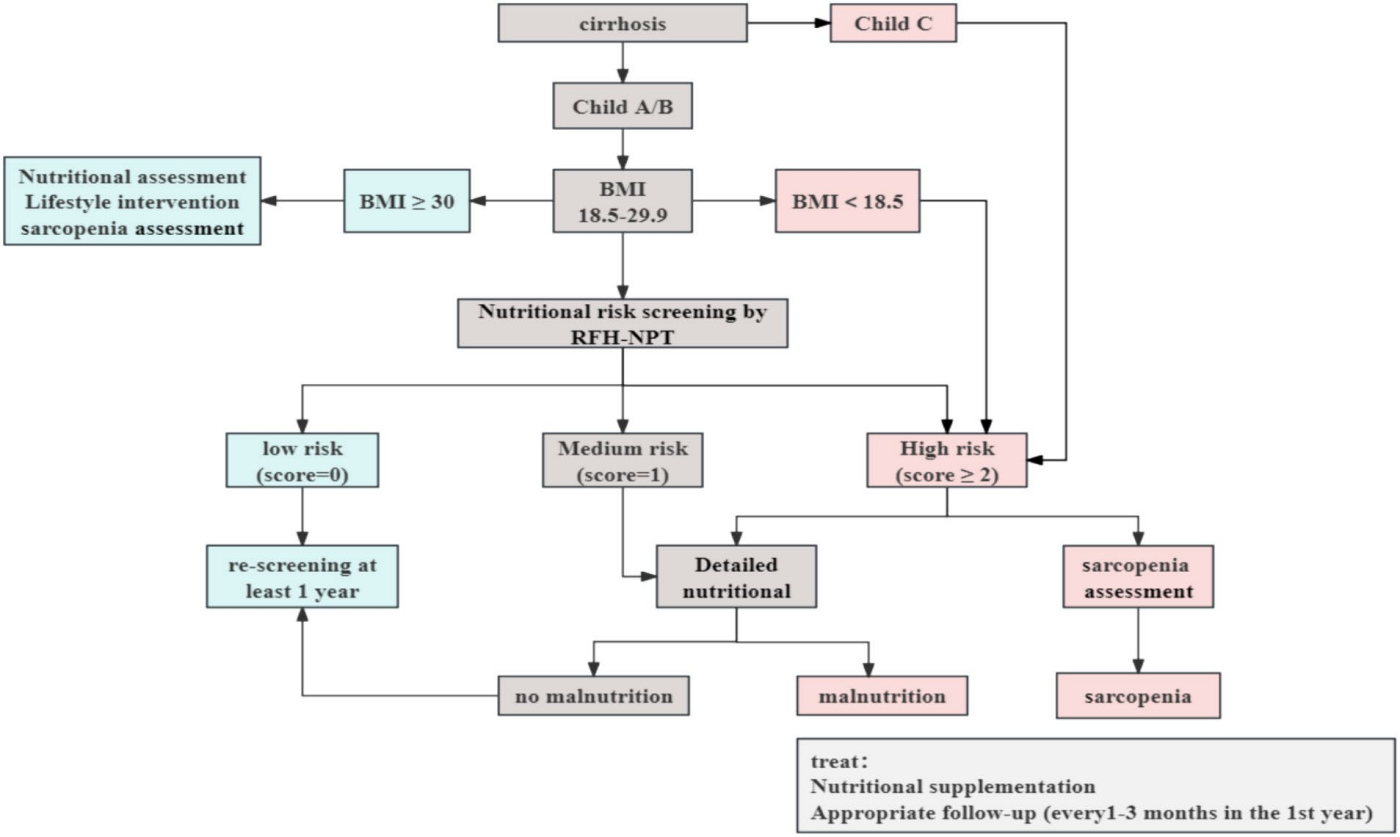
Nutritional screening and assessment in patients with cirrhosis. Adapted from the European Association for the Study of the Liver (EASL) clinical practices guidelines (4), Created with ProcessOn; BMI, body mass index; RFH-NPT, Royal Free Hospital-Nutritional Prioritizing Tool.

According to the European Association for the Study of the Liver (EASL) Clinical Practice Guidelines, Child-Pugh score and Body Mass Index (BMI) should be calculated for all cirrhotic patients presenting to the clinic. Patients with cirrhosis often have sodium and water retention, which interferes with this calculation. The BMI was calculated using “dry weight” for patients with peripheral edema and ascites. For mild, moderate, or severe ascites, the current body weight was decreased by 5, 10% or 15%, respectively; for peripheral edema, another 5% reduction was applied (4). Cirrhotic patients with Child-Pugh C or BMI < 18.5 kg/m^2^ are regarded as high nutritional risk and should undergo a complete nutritional assessment immediately, including an evaluation for sarcopenia as a complication of malnutrition. For obese patients (BMI > 30 kg/m^2^), nutritional and lifestyle interventions targeting obesity are required. For cirrhotic patients with a BMI between 18.5–29.9 kg/m^2^, the effect of fluid retention/ascites on BMI needs to be considered, therefore the use of nutritional risk screening that considers the effects of fluid retention is a prerequisite, and patients at intermediate/high nutritional risk are then subjected to a detailed nutritional assessment. Patients at low risk of malnutrition should be re-screened annually. Cirrhotic patients who are screened at high risk of malnutrition should be assessed and monitored every 1–6 months in the outpatient setting, and hospitalized patients should be assessed and documented on admission and at regular intervals throughout their hospitalization (4, 38). Figure 2 shows a synthesized protocol for screening and assessing malnutrition in liver cirrhosis, which was adapted from the EASL clinical practice guidelines.

However, many patients with cirrhosis nowadays tend to have a normal or even to be obese, so one of the major reasons why BMI does not necessarily reflect nutritional status is the loss of muscle mass. Sarcopenic obesity (Sa-O) refers to the coexistence of sarcopenia and obesity as measured by dual-energy X-ray absorptiometry, and describes the interactions between obesity and sarcopenia that are associated with decreased physical activity and reduced energy expenditure (39). One study found that patients with cirrhosis combined with Sa-O had a worse median survival than patients with normal body composition (40). Sarcopenia is a muscle disease that is defined as a reduction in the quantity, strength, and function of skeletal muscle (41). But the American Association for the Study of Liver Diseases (AASLD) practice guidelines present a consensus definition of sarcopenia in patients with cirrhosis as loss of muscle mass (42). As of 2021, the American Association for the Study of Liver Diseases considers sarcopenia and malnutrition to be interrelated, therefore, sarcopenia is considered to be a major component of malnutrition in cirrhotic patients (1, 42). The prevalence of sarcopenia in patients with cirrhosis is approximately 40–41% (43, 44).

A wide variety of nutritional risk screening and assessment tools are available in clinical practice; this review summarizes the nutritional screening and assessment tools commonly used in clinical practice, as well as the commonly used diagnostic methods for sarcopenia.

## 2 Screening of malnutrition

The widely used nutritional screening tools comprise Nutritional Risk Screening 2002 (NRS-2002), Malnutrition Universal Screening Tool (MUST), Royal Free Hospital-Nutritional Prioritizing Tool (RFH-NPT) and Liver Disease Undernutrition Screening Tool (LDUST).

NRS-2002 is a tool suggested by the ESPEN for screening the indications for nutritional support in hospitalized patients (45). The NRS-2002 intends to identify patients who may benefit from subsequent nutritional care support (46). The NRS-2002 scoring system estimates nutritional impairment, disease severity, and age (47). The three component total scores categorized patients into a no-risk group (< 3 points) and a malnutrition risk group (≥ 3 points). Notably, the NRS-2002 has been demonstrated in several studies to be a reliable predictor of clinical consequences, such as the occurrence of disease complications, prolonged length of hospitalization, and mortality (48). However, it is highlighted that the validity and effectiveness of the NRS-2002 to identify patients at malnutrition risk varies considerably across disease populations and age groups (49). Further research is therefore warranted to elucidate its utility in the context of cirrhosis. This is because cirrhotic patients usually have sodium and water retention, resulting in inaccurate scores.

The MUST was designed by the British Association for Parenteral and Enteral Nutrition to screen for the malnutrition risk of all adult patients (50, 51). It consists of three main components: unintended loss of weight, current BMI, and the existence of any acute illness that may affect nutritional intake for >5 days. Accordingly, the three component total scores categorized patients into a low-risk group (score = 0), medium-risk group (score = 1), or high-risk group (score ≥ 2). In 2015, ESPEN defined malnutrition as a state of altered body composition (reduced fat-free mass) attributable to reduced nutrient intake or absorption, resulting in reduced physical and mental functioning and affected clinical outcomes of disease (52). It has been shown that MUST scores correlate relatively well with the criteria for malnutrition as defined by the ESPEN; However, the sensitivity of MUST is much lower than that of NRS-2002, RFH-NPT, and LDUST (29).

The RFH-NPT was developed for patients with alcoholic cirrhosis; the assessment is divided into three steps. First, patients with acute alcoholic hepatitis or tube feeding are considered to be at high risk immediately. Second, it was evaluated for fluid overload and its impact on food intake and body weight; and third, the nutritional status of patients without fluid overload was assessed on the basis of body mass index, unplanned weight loss, and dietary intake per day. This metric has been verified in a multi-center study in the United Kingdom and is considered an independent predictor of disease progression and survival (53, 54). The RFH-NPT takes into account the impact of sodium and water retention on nutritional screening in cirrhotic patients. However, RFH-NPT was originally developed for patients with alcoholic cirrhosis. In China, where viral cirrhosis is predominant, a prospective study assessing the nutritional status of cirrhotic patients attributable to hepatitis viral infections found that the RFH-NPT detected more patients with decompensated cirrhosis who may be at risk for malnutrition when compared with the NRS-2002 (29). One advantage of RFH-NPT is that it considers the impact of sodium and water retention on scoring, which is usually present in patients with cirrhosis at decompensated stage. Previous studies by our team have determined for the first time the relationship between serum micronutrient concentrations and the risk of malnutrition as assessed by the RFH-NPT in patients with cirrhosis (55). In addition, RFH-NPT is an independent predictor for disease progression. This emphasizes the importance of RFH-NPT to screen cirrhotic patients for malnutrition risk and its implication to predict patient prognosis (54). Taken together, RFH-NPT appears to be more valuable for nutritional risk screening in cirrhotic patients (56, 57).

LDUST has been identified for use in patients with cirrhosis. It was developed by the American Society for Parenteral and Enteral Nutrition and the Academy of Nutrition and Dietetics with limited available data in China. The LDUST comprises a total of six questions, suggestive of loss of weight, food intake, muscular loss, edema or fluids, and daily activities where nutritional grading is based on the final score; a score of 5 or more is graded as A and determined to be no risk; 2–5 is graded as B, < 2 is graded as C, and grades B and C are at risk for malnutrition (58). Since the assessment component of LDUST relies in part on the subjective judgment of the patient, this can lead to bias. LDUST has some limitations. Previous data indicate that LDUST has a relatively high positive and a relatively low negative predictive value for cirrhotic patients, thus some investigators consider it a negative screening tool that does not reliably identify patients with malnutrition (29). A study has shown that NRS2002 and RFH-NPT were superior to LDUST at detecting the malnutrition in cirrhosis patients diagnosed according to GLIM criteria (59).

Mini Nutritional Assessment-Short Form (MNA-SF) is mainly used in elderly patients and contains two components. Some studies have shown that MNA-SF has high sensitivity and specificity (60). Although it has been shown that MNA-SF can be used for nutritional risk screening in patients with cirrhosis (61), there are fewer relevant studies, and more studies are needed to validate MNA-SF for nutritional risk screening in cirrhotic patients. Simplified Nutritional Appetite Questionnaire (SNAQ) is mainly used to screen elderly patients for malnutrition due to decreased appetite. In cirrhotic patients, decreased appetite and intake due to ascites, portal hypertension, and salt restriction is one of the major causes of malnutrition in these patients. Therefore, it has been shown that SNAQ can be used to evaluate decreased appetite and predict weight loss in cirrhotic patients (62). Nutrition Risk in Critically ill (NUTRIC) is used to assess the nutritional risk of patients in the intensive care unit (ICU) and is used for early identification of patients most likely to benefit from intensive nutritional support. The score incorporates the variables age, comorbidities, days from admission to transfer to the ICU, Acute Physiology and Chronic Health Evaluation II (APACHE II), Sequential Organ Failure Assessment (SOFA), and interleukin 6 (IL-6) (60). A study has demonstrated the high prognostic accuracy of NUTRIC in critically ill patients with cirrhosis (63). However, there are few reliable data on nutritional risk assessment in critically ill patients with cirrhosis.

## 3 Assessment of malnutrition

Commonly used nutritional assessment tools include Subjective Global Assessment (SGA), Patient-Generated Subjective Global Assessment (PG-SGA) and GLIM criteria.

The SGA questionnaire serves as the most widely used nutritional assessment tool in clinical (56), and SGA is one of the tools recommended by ESPEN and EASL for nutritional assessment of patients with liver disease (4, 64). SGA includes the following aspects: Weight loss, unintended reduction in dietary intake, gastrointestinal dysfunction, body functions, diseases and their relationship to nutritional needs, loss of muscle and fat mass, and fluid retention. Good nutritional status is graded A, moderate malnutrition is graded B, and severe malnutrition is graded C (65). One study showed that SGA-rated malnutrition was associated with increased number of unplanned hospital admissions (66). Other studies have also implicated a correlation between malnutrition and mortality in cirrhotic patients assessed by SGA (1, 67). However, more researches are needed to support the use of SGA in cirrhotic patients, because there are some limitations to the use of SGA, such as underestimation of the prevalence of sarcopenia (1, 67, 68).

The PG-SGA is a modified version of the nutritional assessment tool SGA. The PG-SGA consists of two parts, the first is a patient self-assessment including weight change, symptoms, functional capacity, and food intake; and the second is completed by both the professional and the patient including comorbidities, metabolic stress, and physical examination (69). The PG-SGA is a validated nutritional assessment tool recommended by ESPEN (70). Initially PG-SGA was used primarily in patients with tumors, PG-SGA has been validated in a wide range of patient populations and is often characterized as the “gold standard” for malnutrition diagnosis (69). However, cirrhotic patients usually experience sodium and water retention, which may affect correct judgment of weight change. The development of malnutrition is usually a long-term process, and the component within PG-SGA regarding unintentional weight loss covers a time frame of more than 1 month, which may interfere with the assessment. Additionally, weight change, nutritional impact symptoms, food intake, and physical functioning in SGA and PG-SGA may contribute to recall bias. The Royal Free Hospital Global Assessment (RFH-GA) was also derived from the SGA and is primarily used to determine the nutritional status of cirrhotic patients. However, this approach is time consuming and requires trained personnel to obtain consistent results, which limits its broad usage (15, 18).

In 2018, the GLIM reached a consensus on the diagnostic criteria for malnutrition and was proposed as the international consensus standard for diagnosing malnutrition (71). The GLIM consensus recommends that nutritional status be assessed on the basis of phenotypic criteria (low body mass index, unintentional weight loss, and loss of muscle mass) in combination with etiologic criteria (reduced intake or assimilation, and disease or inflammatory conditions); at least one of the phenotypic and one of the etiologic criteria must be present in order to make a diagnosis of malnutrition. A meta-analysis showed that the GLIM criteria have high diagnostic accuracy in differentiating malnutrition and have the potential to become the gold standard for diagnosing malnutrition in clinical practice (72). Malnutrition as defined by GLIM was associated with significantly higher in-hospital mortality and poor clinical outcomes (72, 73). It is worth noting that there is no uniformity in the GLIM diagnostic criteria for loss of muscle mass. Suggested methods of muscle mass assessment include bioelectrical impedance, ultrasound, dual-energy absorptiometry, CT, MRI, or other measurements such as calf muscle circumference or mid-arm muscle circumference (MAMC), as well as handgrip strength (HGS) as an ancillary measure (71). Some studies have also used fat-free mass index as an alternative measurement (74).

In addition to nutritional assessment using the Nutritional Assessment Tool, anthropometric, body composition analysis and laboratory indicators can also be used to assess the nutritional status of patients with cirrhosis. The main anthropometric indicators are Arm Circumference (AC), Triceps Skinfold (TSF) and Mid-Arm Muscle Circumference (MAMC). These indices are easy to perform and are effective methods of nutritional assessment in patients with liver disease at the bedside. AC, TSF and MAMC are more commonly used in nutritional assessment because they can be measured directly and are simple to perform, and are not affected by the patient’s sodium and water retention. AC and TSF are sensitive indices of the patient’s muscle and fat reserves. The cut-off value of AC for the diagnosis of malnutrition is 26 cm in men and women. The reference value of TSF is 8.3 mm for men and 15.3 mm for women, and the reference value of MAMC (MAMC = AC-3.14*TSF) is 24.8 cm for men and 21.0 cm for women. TSF and MAMC are used to determine malnutrition based on the percentage of the normal reference value, i.e., > 90% of the measured value/normal value is considered normal, and 80–90% is considered mild malnutrition; Between 60–80% is considered moderate malnutrition; < 60% is considered severe malnutrition. Commonly used laboratory indicators are mainly albumin and prealbumin, which reflect the function of hepatic synthesis; albumin is more affected by exogenous supplementation, so albumin is of low value in assessing the nutritional status of cirrhosis. Prealbumin changes are more sensitive than albumin, and prealbumin can still be synthesized during the decompensated phase of cirrhosis, whether prealbumin can be used as a measurement of malnutrition in cirrhosis remains to be studied (75).

The most researched and widely used body composition analysis is Bioelectrical Impedance Analysis (BIA), which is based on the principle of calculating the impedance, i.e., the electrical resistance of body water and the reactance of cell mass, by the conduction of electrical currents through the body, in order to estimate the measurements of body composition (76). The BIA includes nutritional indicators such as phaseangle (PA), skeletal muscle content, body fat mass, body fat percentage, extracellular water ratio, and other nutritional indices. The advantages of BIA are that the results are easy to obtain, are less affected by sodium and water retention, correlate well with liver function scores, and are more accurate in patients with cirrhosis who do not have sodium and water retention; however, BIA should not be performed in patients with a history of pacemaker or defibrillator implantation and amputation (77). PA is the magnitude of the change in AC phase in response to cell membranes in the human body and is based on the reactance and impedance values generated by the body. PA increases when the cell membrane structure is intact and function increases, and decreases when the cell membrane structure is damaged or selective osmotic function decreases. PA reflects the amount of cells in the body and the integrity of the cell membrane structure and physiological function, and can be used as an indicator of nutritional judgment. In a research study, PA ≤ 4.9 was found to be a predictor of death in patients with cirrhosis, and PA is a useful and reliable tool for evaluating the prognosis of cirrhosis (78).

## 4 Sarcopenia

In patients with cirrhosis, malnutrition is characterized by depletion of skeletal muscle and adipose tissue mass, with the main nutritional consequences of the loss of skeletal muscle mass (79). Sarcopenia can be measured by handgrip strength (HGS) in addition to the Skeletal Muscle Index (SMI) (41). However, many factors affect HGS, patient’s age, occupation may affect HGS. Therefore, the diagnosis of sarcopenia using HGS may be biased, so many studies typically use SMI to diagnose sarcopenia. SMI was expressed as the skeletal muscle area at the L3 or T12 level divided by the height squared (cm^2^/m^2^). There are differences in the cut-off values for differentiating sarcopenia in different countries and regions. Sarcopenia was defined as a SMI ≤52.4 cm^2^/m^2^ in male patients and SMI ≤38.5 cm^2^/m^2^ in female patients (80). However, this data is mainly derived from European and American populations. A study in China indicated 44.77 cm^2^/m^2^ for male patients and 32.50 cm^2^/m^2^ for female patients as the cut-off values for L3-SMI (81). Japanese scholars have defined sarcopenia in liver disease patients under 65 years of age as SMI < 42 cm^2^/m^2^ in men and SMI < 38 cm^2^/m^2^ in women (82). Although there are a variety of studies pertinent to sarcopenia in patients with liver disease, there is no standardized SMI criteria for diagnosing sarcopenia. Strong correlations have been demonstrated between individual cross-sectional magnetic resonance imaging (MRI) or computed tomography (CT) data and body composition (83). L3-SMI is the ratio of the cross-sectional area of the lumbar major muscle at the third lumbar vertebrae to the square of height on CT or MRI. L3-SMI has been identified for the quantitative assessment of loss of muscle mass and recognized as an objective, quantifiable parameter that can be used to assess nutritional status (81). It is a quantitative, objective, non-invasive, and simple method and is considered to be the gold standard for the assessment of sarcopenia in the context of cirrhosis (4, 84). Although it is costly to perform CT specifically to calculate SMI, patients are exposed to unnecessary radiation. Notably, patients with cirrhosis often undergo CT for other reasons (e.g., to screen for hepatocellular carcinoma), so this approach is clinically feasible; and SMI values calculated from thoracic spine 12 (T12) levels also showed a correlation with mortality (41, 79). Therefore, CT images at the T12 level can be used to calculate SMI for those patients who do not have abdominal CT. A review indicated that ultrasound testing for sarcopenia in patients with advanced liver disease is safe, feasible, and shows good correlation with gold standard measurements of sarcopenia and can be used as a valid tool in daily practice (85). The use of ultrasound for the evaluation of sarcopenia also has a number of limitations such as those related to the type of probe used (linear or convex), the anatomical site of measurement, the patient’s posture during the examination, the position of the probe, the pressure exerted by the probe, and the type of parameters obtained (86). The strength, assistance walking, rise from a chair, climb stairs, and falls (SARC-F) questionnaire is a well-established tool for screening for sarcopenia and sarcopenia-related dysfunction. The SARC-F score also has good sensitivity as a bedside screening tool for sarcopenia in cirrhotic patients. Cirrhotic patients with high SARC-F scores and low MAMC require further evaluation for sarcopenia (87).

Sarcopenia is associated with poor prognosis and reduced survival rate before and after liver transplantation (88). Cirrhotic patients with sarcopenia were prone to experience worse prognosis and a significantly higher mortality rate when compared to those without sarcopenia (89, 90). Furthermore, the presence of sarcopenia is closely associated with the development of complications in patients with cirrhosis, such as ascites, esophageal varices, and hepatic encephalopathy (91). According to previous studies, sarcopenia increases the risk of ascites more triple fold, and cirrhotic patients with sarcopenia have a much higher risk of developing hepatocellular carcinoma (90, 92). Frailty, also very common in patients with cirrhosis, is a multidimensional concept that represents the ultimate manifestation of disorders of multiple physiologic systems, resulting in reduced physiologic reserves and increased vulnerability to health stressors (93). Frailty, malnutrition and sarcopenia overlap with each other in patients with cirrhosis, and there is a lack of evidence on whether the assessment of weakness contributes to the assessment of nutritional status in patients with cirrhosis.

## 5 Summary

Malnutrition is a crucial complication in patients with liver cirrhosis and is associated with the occurrence, development and deterioration of other complications. Nutritional interventions for these patients can curtail the complication and mortality to a certain extent and improve the quality of life among cirrhosis. Therefore, we need to identify malnourished patients promptly and accurately. RFH-NPT is a more feasible tool to screen cirrhotic patients for nutritional risk, and is highly reproducible, and considers the impact of sodium and water retention, thus making it practical to screen cirrhotic patients. Subsequently, GLIM diagnostic criteria may be used to evaluate the nutritional status of patients with nutritional risk via the GLIM diagnostic criteria.

L3-SMI can accurately define sarcopenia in cirrhotic patients and also be used for clinical nutritional status assessment. For malnourished patients identified by dietitians according to conditions of the patients to tailor specific nutrition program, regular follow-up, and timely adjustment of nutrition program.

## 6 Recommendations

Although studies have confirmed that RFH-NPT is suitable for nutritional risk screening in patients with viral cirrhosis, there is paucity of data available. Hopefully, more data will be available in the future to support this conclusion. Although the GLIM diagnostic criteria for malnutrition have been shown to be relatively accurate in identifying malnourished cirrhotic patients, this may lead to errors of judgment due to the ambiguity of the thresholds for the phenotypic criteria, especially for reduced muscle mass. This is expected to be followed by more studies in the future to propose a harmonized diagnostic index for the GLIM diagnostic criteria. In addition, although L3-SMI has been proved to be used to evaluate sarcopenia, some studies have proposed that L3-SMI combined with HGS is more accurate in evaluating sarcopenia, and HGS is affected by many factors. More studies will hopefully be conducted in the future to propose more objective assessment criteria.

## Author contributions

YH: Writing – original draft, Writing – review & editing. ZW: Writing – original draft, Writing – review & editing. SW: Writing – review & editing. LL: Writing – review & editing. JL: Writing – review & editing. YZ: Writing – review & editing. BC: Writing – review & editing. XS: Writing – review & editing. CS: Writing – review & editing. LW: Writing – review & editing.
